# Evidence and alternatives to the routine use of cuffed tracheostomy tubes in spontaneously breathing patients with neurological disorders

**DOI:** 10.3389/fresc.2026.1737845

**Published:** 2026-02-25

**Authors:** Bettina Arca-Tschudi, Monika Rüegg, Paul Diesener, Karsten Krakow

**Affiliations:** 1 Zihlschlacht Neurological Rehabilitation Clinic, Zihlschlacht, Switzerland; 2 Private University in the Principality of Liechtenstein (UFL), Triesen, Principality of Liechtenstein; 3 Private Speech Therapy Office, Winterthur, Switzerland; 4 Department for Neurological Rehabilitation, Hegau-Jugendwerk, Gailingen, Germany

**Keywords:** aspiration, cuffed tracheostomy tube, evidence based tracheostomy tube management, neurological rehabilitation, non ventilated, spontaneous breathing, tracheostomy tube weaning, tracheotomized

## Abstract

**Introduction:**

Cuffed Tracheostomy tubes (TT) are often used in spontaneously breathing patients with neurological impairment because of a high risk of aspiration. Besides to the well-known risks, cuffed TT impair cough efficiency and therapeutic strategies for the treatment of dysphagia.

**Methods:**

A PRISMA-based systematic review of MEDLINE, PubMed and Cochrane examined the available evidence on cuffed tracheostomy tubes in spontaneously breathing, neurologically impaired adult patients and summarized key findings and alternatives to their routine use.

**Results:**

The studies—mostly clinical reports and narrative reviews—describe in-house treatment strategies to minimize tracheal damage caused by cuffed TT. No RCTs were found that prove the benefit of cuffed TT in patients with a high risk of aspiration.

**Conclusion:**

The current data does not support the routine use of cuffed TT in non-ventilated patients with neurological impairment and a high risk of aspiration. Prospective studies are required to compare the benefit of cuffed vs. cuffless TT in this population to prevent aspiration pneumonia.

## 1 Introduction

Tracheostomy management in neurological rehabilitation has gained increasing attention in recent years, as these patients often remain tracheotomized for extended periods and present complex interactions between respiration, swallowing, and communication. If patients are ventilated or have tracheal instability, the use of cuffed TT is essential for survival (1). However, in neurologically impaired adults who breathe spontaneously (e.g., after a stroke, traumatic brain injury or in patients with neurodegenerative diseases), the benefit of cuffed TT, regarding mortality, morbidity, and quality of life (QoL) is not proven. Despite this clinical relevance, current guidelines provide no specific recommendations regarding cuff use in spontaneously breathing adults, offering instead only broad and often unspecific statements (2). In clinical practice, however, the authors observe that cuffs are frequently kept inflated until decannulation, despite the lack of evidence-based justification for this approach.

The presumed protection against aspiration and the undisputed advantages of cuffed TT, such as splinting of an unstable trachea and the option of ventilation on the one hand, and disadvantages, such as mucosal ischemia, tracheal collapse due to cartilage malacia or dilation of the pars membranacea, impaired cough efficiency and aphonia as well as impaired swallowing therapy on the other hand, have not yet been sufficiently weighed. The management of tracheostomy tubes varies between hospitals because of the lack of evidence-based guidelines (3).

The present review aims to synthesize the available evidence on the advantages and disadvantages of cuffed tracheostomy tubes in spontaneously breathing adults with neurological impairments and to critically examine the role of alternative tracheostomy management strategies in this population.

## 2 Methods

**Figure 1:**
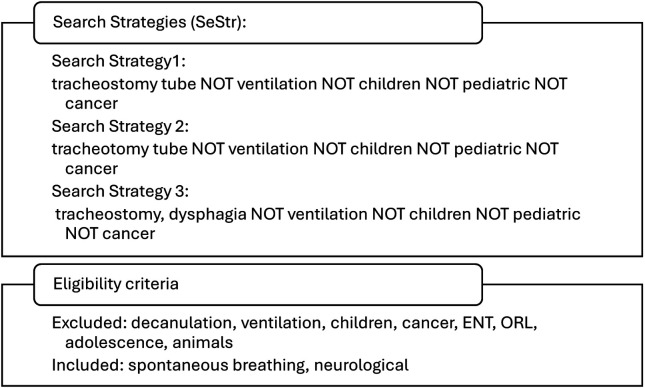
Search strategy.

The search strategy comprised a comprehensive review of MEDLINE, PubMed, and the Cochrane Library with the objective of identifying studies focusing on adult neurological patients who are spontaneously breathing and have a tracheostomy tube in place (Table 1). The search was concluded in August 2025, and the documentation thereof was carried out in accordance with the PRISMA protocol (4). PRISMA (Preferred Reporting Items for Systematic Reviews and Meta-Analyses) principles were used in a simplified manner to enhance transparency in the literature selection process of this review. The full search syntax and Boolean combinations are provided in Figure 1. Key search terms included: tracheostomy; tracheostomy tube; cuffed; cuffless; fenestrated; speaking valve; capping; decannulation; downsizing; neurogenic dysphagia; stroke; traumatic brain injury; neurodegenerative disease.

**Table 1:** Main characteristics of the studies included in the review.

Study	Design	Findings
Berges et al. 2022: Impact of Low-Volume, Low-Pressure Tracheostomy Cuffs on Acute Mucosal Injury in Swine (5)	*In-Vivo* Animal experimental study	All cuffs caused mucosal injuries in the trachea
Cooper JD 2018: Tracheal Injuries Complicating Prolonged Intubation and Tracheostomy (6)	Narrative review	Documents injuries because of mispositioning and overinflated cuffs
Corbett et al. 2020: x-Ray and CT Scan Based Prediction of Best Fit Tracheostomy Tube—A Pilot Study (17)	Retrospective observational cohort study	Imaging can help select appropriate tube size and reduces the risk of cuff related complications
Freeman-Sanderson et al. 2018: Quality of Life Improves for Tracheostomy Patients with Return of Voice (13)	Mixed methods	Early restoration of voice function with speaking valves, fenestration and cuff deflation significantly improves quality of life
Hernandez et al. 2012: The effects of increasing effective airway diameter on weaning from mechanical ventilation in tracheostomized patients: a randomized controlled trial (10)	Randomized controlled trial	Deflating the tracheal cuff during weaning leads to faster weaning from mechanical ventilation reduced respiratory infections, improved swallowing function, sufficient cough (secretion clearance)
Kim et al. 2017: Effects of Capping of the Tracheostomy Tube in Stroke Patients With Dysphagia (12)	Clinical study	Significantly reduced vallecular residue with capped tube
Kowalski et al. 2017: Assessment of Cough Strength in Patients With Tracheostomies (9)	Randomized crossover study	Shows improved cough and better secretion clearance after cuff deflation and with use of speaking valves
Otto et al. (2025) Plea for routine endoscopic tracheostomy tube adjustment (8)	retrospective single-center analysis	Mispositioned tracheostomy tubes in 65% of examinations, mucosal injuries detected in 19%, correct positioning in 35%.
Pandian et al. 2017: Predicting the Need for Nonstandard Tracheostomy Tubes in Critically Ill Patients (7)	Matched case-control study	Importance of selecting nonstandard tubes when individual anatomy or clinical situation requires
Pandian et al. 2019: Are Fenestrated Tracheostomy Tubes Still Valuable? (16)	Retrospective chart review (2007–2017)	Fenestrated tubes are beneficial for voice production if they fit well (rare cases)
Park & Lee 2018: Changes in Swallowing and Cough Functions Among Stroke Patients Before and After Tracheostomy Decannulation (11)	Prospective observational study	Swallowing improved after tracheostomy tube removal, better prognosis for patients with successful decannulation
Pryor et al. 2016: Clinical indicators associated with successful tracheostomy cuff deflation (14)	retrospective case-note and chart audit	95% of patients tolerated first cuff-deflation
Pryor et al. 2016: Patterns of return to oral intake and decannulation post-tracheostomy across clinical populations (15)	Retrospective review	Continuous deflation of the cuff after 7.5 days, commencement of oral food intake after 10.5 days, decannulation after 15 days. The majority started oral intake after cuff deflation or with uncuffed tube
Wignall & Baines 2014: The effect of cuff presence and cuff inflation on airway pressure in a canine tracheostomy tube model (18)	Ex-Vivo experimental study	The presence of a cuff significantly increases airway pressure

We considered publications spanning all available years. Eligible manuscripts were those published in languages accessible to the review team; primary languages searched were English and German. Studies in other languages were considered when an English abstract or reliable translation was available.

**Figure 2:**
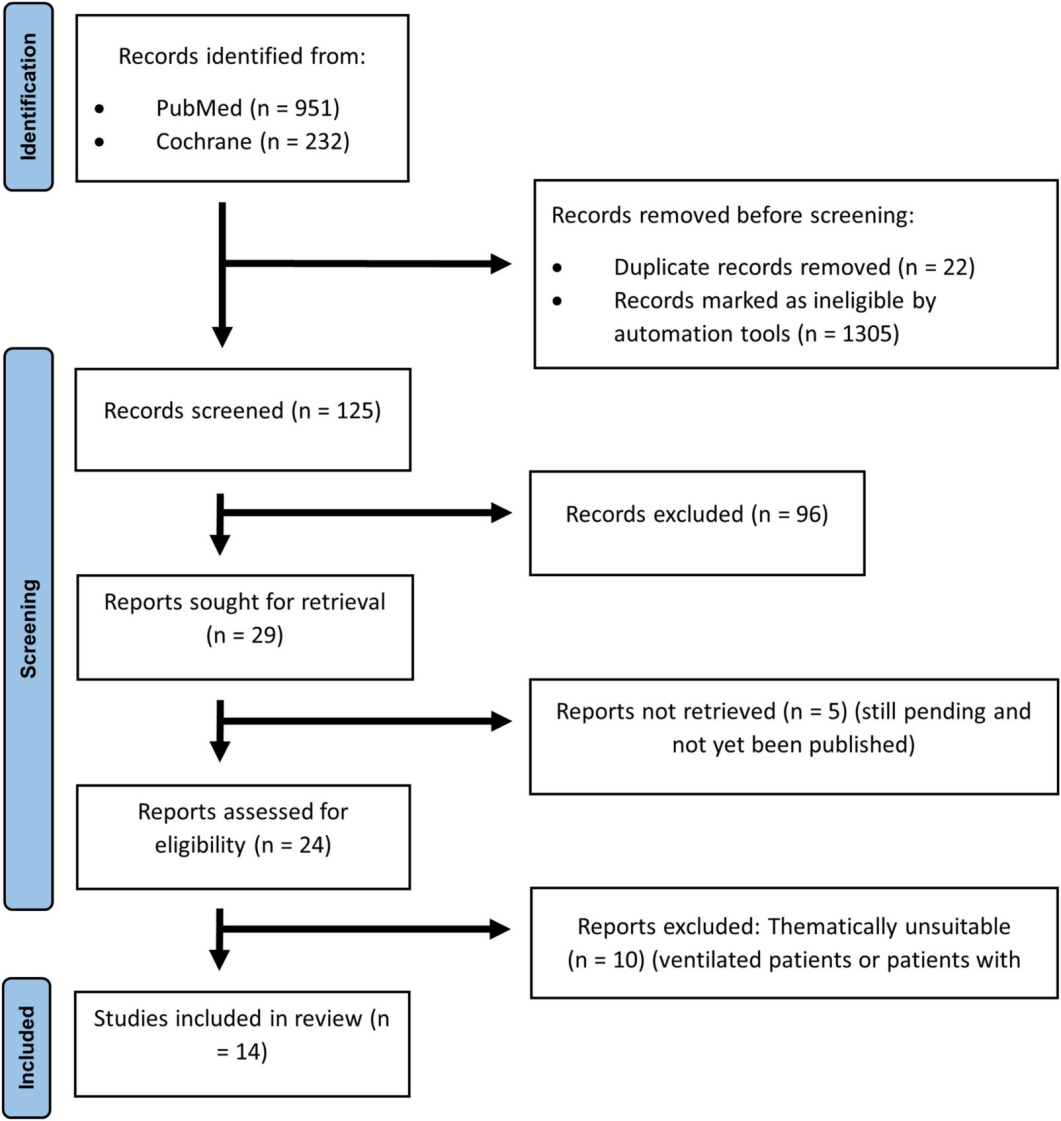
PRISMA flow diagram showing the study selection process (4).

A total of 125 records were screened, 29 full text articles were assessed for eligibility, and 14 studies were included in the final synthesis (see Figure 2). The included studies were published between 2012 and 2025. Detailed study characteristics and quality assessments are provided in Table 1.

To be considered for inclusion in the present review, research studies had to include adult patients with neurological impairment who had undergone a tracheotomy and were able to breathe spontaneously without the requirement of continuous invasive positive pressure ventilation. Studies involving children were excluded from the analysis. The following interventions or exposures were considered: the presence of a cuffed tracheostomy tube or a cuffless tube; the use of cuffless or fenestrated tubes; studies with capping or speaking valves; and tube downsizing.

The primary outcomes of interest included cough strength, swallowing function and saliva aspiration, voice recovery, tracheal injuries or complications such as granulomas, stenosis and dilation, the success of decannulation, and QoL, as defined in the guideline “neurogenic dysphagia” (2).

The types of studies included in the review were crossover randomized controlled trials, controlled clinical trials, clinical observational studies, retrospective reviews, case-control studies, laboratory or animal studies, and narrative reviews. The studies were reviewed based on the criteria described above and selected for inclusion.

## 3 Results

The extant literature contains no evidence to suggest that inflating a cuff in spontaneously breathing patients with neurogenic dysphagia and high risk of aspiration improves outcome parameters such as survival, reduction in aspiration pneumonia or faster rehabilitation. In instance of benefits being claimed (e.g., protection of the airways), these benefits are theoretical in nature or limited to patients who require assisted ventilation or have rare conditions that cannot be treated by other means (e.g., gastroparesis with bile reflux, cholinergic crisis or uncontrollable vomiting).

There is evidence to suggest that the use of cuffs can result in damage to the trachea. An *in-vivo* animal study and a narrative review show that cuff pressure causes mucosal ischemia, ulceration and stenosis or dilation. Poor size selection, mispositioned tubes, or chronically overinflated cuffs have been demonstrated to increase the risk of injuries (2, 5). The significance of individual diagnostics and the accurate selection of the tracheal cannula size is underscored in the literature (6). Furthermore, the correct position and size should be clarified using imaging techniques (7).

A number of clinical studies have emphasized improved cough strength (8), faster weaning from mechanical ventilation (9), and earlier voice and swallowing function after cuff deflation, downsizing or removal (10–13). The early restoration of functions is associated with an enhancement of QoL (13). The evaluation of patients' readiness for cuff deflation is supported by established indicators and protocols (14) and many patients tolerate early deflation well (14). Capping and speaking valves have been found to enhance cough, phonation and QoL in many cases. Furthermore, research has indicated that these valves can reduce aspiration residues (9, 11, 13).

Retrospective data support the hypothesis that the utilization of suitable fenestrated tubes is beneficial, when voice function is a priority (13) and no granulations occur because the cannula is correctly positioned in the trachea (15). The practice of downsizing prior to decannulation is a common procedure and is associated with enhanced swallowing function and an increased probability of successful decannulation (10, 11, 14). The selection of the most appropriate tube is dependent on the imaging modality (x-ray/CT or tracheoscopy) employed for guidance, with the objective of ensuring a suitable fit and averting issues that could lead to elevated cuff-related risks (16). There is evidence to suggest that, in any case, the use of cuffs can result in damage to the trachea (2) and an increase in airway pressure (17).

The included studies demonstrate significant heterogeneity in terms of study design and population. The literature under review here includes clinical trials, ex-vivo studies, retrospective reviews, small crossover studies and qualitative reports. The predominance of small pilot studies and observational studies suggests a considerable risk of publication bias.

## 4 Discussion

The existing body of evidence offers no high-quality data demonstrating that the maintenance of an inflated tracheostomy cuff in spontaneously breathing patients with neurological diseases achieves clinically significant better outcomes, such as survival, reduction in aspiration pneumonia or accelerated rehabilitation. When potential benefits are described, they tend to be theoretical or apply only to specific subgroups requiring positive pressure ventilation or exhibiting unusual symptoms, such as uncontrollable vomiting or severe reflux.

A substantial body of research has documented the occurrence of cuff-related damage, employing diverse research methodologies, including *in vivo* and *ex vivo* animal models (2, 17), as well as narrative reviews (5). The damages comprise mucosal ischaemia, elevated airway pressure, ulcerations and long-term structural damage to the trachea associated with cuff pressure. Inadequate tube selection, mispositioned tubes and persistent overinflation have been identified as factors that heighten the probability of complications (5, 6). In order to mitigate the potential for complications, it is recommended that imaging be used to guide the selection of cannula type and size, and accurate positioning of the tracheostomy tube, and that individualised treatment planning be performed (6, 16).

The deflation of cuffs, the downsizing of tubes, and the utilisation of cuffless or well-fitting fenestrated designs have been found to result in improvements in functional outcomes, including cough strength, swallowing ability and voice production (8, 9, 15). Several clinical studies have reported that early deflation of the cuffs is commonly well tolerated. This has been observed to facilitate the restoration of airway protective functions, reduce residues, and lead to successful decannulation (10, 14, 15). The restoration of swallowing and speech function in the early stages of treatment has been identified as a significant contributor to QoL, as evidenced by studies examining speaking valves and capping strategies (10, 13). This approach contributes to the restoration of communication skills, enhances cough efficiency, and reduces secretion burden, thereby promoting both rehabilitation and patient autonomy (8, 9). The individual assessment of the readiness for cuff deflation using validated indicators must be considered an essential part of this process. A range of protocols have been developed to support clinical teams in their decision-making processes (14). The utilisation of imaging modalities, such as endoscopic control, x-ray and CT, facilitates individualised tube fitting and thereby minimises the occurrence of complications (16). Fenestrated tubes may offer additional benefits for phonation in selected patients, provided that the risk of granuloma formation remains low through careful placement and monitoring (15).

Nevertheless, the corpus of literature on this subject is limited in scope and significance due to methodological heterogeneity. A considerable number of studies encompass a variety of clinical populations, extending beyond neurological and spontaneously breathing patients, which complicates the interpretation and summarisation of the results for this specific group. The variability in the definitions of outcomes, for example regarding aspiration or tracheal injuries, further restricts the comparability of the studies. The preponderance of available data is derived from small-scale pilot or observational studies, a factor that gives rise to the potential for publication bias. Therefore, it is advisable to exercise caution when directly extrapolating the results to broader clinical practice.

Notwithstanding the limitations previously mentioned, a consistent clinical message can be deduced from the available evidence. The routine practice of cuff inflation in patients with neurological conditions who are breathing spontaneously has not been demonstrated to offer any proven benefit. Moreover, there exists a possibility that this procedure may be associated with measurable risks. It is recommended that clinical management strategies should focus on the early assessment of possible cuff deflation, the use of cuffless or fenestrated tubes, and careful selection and sizing of the tube. Multidisciplinary decision-making and transparent documentation of risk-benefit considerations are essential. Furthermore, it is also important to inform patients and their families about the trade-offs between airway protection, communication, swallowing function, and QoL.

In order to generate a more robust evidence base, there is a necessity for high-quality prospective studies, randomised trials (where ethical considerations permit) and implementation research on standardised protocols. The primary focus of these studies should be on meaningful patient outcomes, including the preservation of intact tracheal mucosa, the prevention of aspiration and pneumonia, the efficacy of cough, the QoL, and the successful decannulation. The development of evidence-based guidelines to ensure safe and effective tracheostomy management in spontaneously breathing neurological patients is an anticipated outcome of the research.

## 6 Conclusion

Current evidence does not support the routine use of cuffed tracheostomy tubes in spontaneously breathing adults with neurological impairment and high risk of aspiration. Potential benefits in this regard remain only presumptive, whereas cuff-related complications and negative effects on swallowing, communication and rehabilitation are well documented. Early cuff deflation, the use of cuffless or appropriately fitted fenestrated tubes, and individualised tube selection appear to support better functional outcomes. In neurological rehabilitation, where the restoration of communication, coughing and swallowing is central to recovery, prolonged cuff inflation may hinder key therapeutic goals and delay functional progress. Given the methodological limitations of existing studies, high-quality prospective research focusing on clinically meaningful endpoints is urgently needed to inform evidence-based guidelines and optimise tracheostomy management in this patient population.
